# Supplementary material for: Enhancing Photoelectric Powder Deposition of Polymers by Charge Control Substances
Source: Polymers (Basel). 2022 Mar 25;14(7):1332. doi: 10.3390/polym14071332 (PMC9002572; doi:10.3390/polym14071332)
Supplement: Supplementary file 1 [file polymers-14-01332-s001.zip › polymers-1655193-supplementary.pdf]

Table S1. Measured averaged peak values of the degree of coverage on the transfer roll

Formulation (cf. Tab. 2)	Silica(-) wt. %	Silica(+) Wt. %	Electrostatic surface potential / V
1.1	0.05	-	-73.0 ± 16.5
2	0.1	-	-61.6 ± 5.8
3	0.25	-	-125.6 ± 4.2
4	0.5	-	-124.9 ± 24.3
5.1	1.0	-	-75.4 ± 5,9
6.1	-	0.05	39.1 ± 10.9
7	-	0.1	54.4 ± 17.2
8	-	0.25	86.5 ± 37.5
9	-	0.5	104.1 ± 34.5
10.1	-	1.0	85.0 ± 27.6

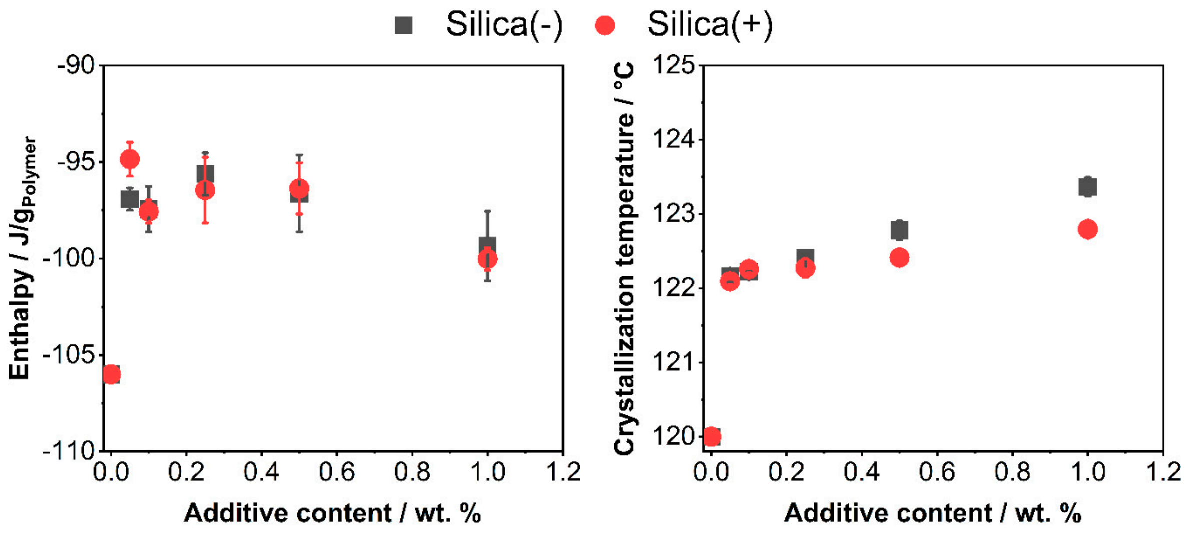

Figure S1. Crystallization behaviour of the functionalized powders; left: Crystallization enthalpy; right: Crystallization temperature; n = 3. The corresponding values are displayed in Tab. 3.
